# BioGraph: Data Model for Linking and Querying Diverse Biological Metadata

**DOI:** 10.3390/ijms24086954

**Published:** 2023-04-09

**Authors:** Aleksandar N. Veljković, Yuriy L. Orlov, Nenad S. Mitić

**Affiliations:** 1 Faculty of Mathematics, University of Belgrade, Studentski trg 16, 11158 Belgrade, Serbia; aleksandar.veljkovic@matf.bg.ac.rs (A.N.V.);; 2 The Digital Health Institute, I.M. Sechenov First Moscow State Medical University of the Ministry of Health of the Russian Federation (Sechenov University), 119991 Moscow, Russia; 3 Institute of Cytology and Genetics SB RAS, 630090 Novosibirsk, Russia; 4 Agrarian and Technological Institute, Peoples’ Friendship University of Russia, 117198 Moscow, Russia

**Keywords:** gene network, associations with the diseases, connecting biological data, BioGraph, metadata, query data properties

## Abstract

Studying the association of gene function, diseases, and regulatory gene network reconstruction demands data compatibility. Data from different databases follow distinct schemas and are accessible in heterogenic ways. Although the experiments differ, data may still be related to the same biological entities. Some entities may not be strictly biological, such as geolocations of habitats or paper references, but they provide a broader context for other entities. The same entities from different datasets can share similar properties, which may or may not be found within other datasets. Joint, simultaneous data fetching from multiple data sources is complicated for the end-user or, in many cases, unsupported and inefficient due to differences in data structures and ways of accessing the data. We propose BioGraph—a new model that enables connecting and retrieving information from the linked biological data that originated from diverse datasets. We have tested the model on metadata collected from five diverse public datasets and successfully constructed a knowledge graph containing more than 17 million model objects, of which 2.5 million are individual biological entity objects. The model enables the selection of complex patterns and retrieval of matched results that can be discovered only by joining the data from multiple sources.

## 1. Introduction

Biological data are highly diverse. Data produced from protein crystallization experiments are very different from those from protein disorder experiments. However, both experiments may give information about the exact biological entities, in this case, the same proteins. As the proteins are sourced from genes, experiments related to their respective genes can also supply valuable information in a broader picture when linked with the protein data. The data are currently stored in isolated data silos, using different formats and are accessible through diverse querying options. Data from various datasets or databases can be linked into a network by matching the common properties. However, a protein record from one database may not contain an exact property that connects it to its respective gene from the other database, but possibly requires a third database to establish that connection.

Some databases, such as MobiDB [1], contain a wide range of entity identifiers sourced from several databases, but the search is based only on exact property matching, without the ability to create complex queries using various metadata attributes. A practical example of a complex query over multiple databases would be selecting human tumor antigen genes associated with proteins with disorder content higher than a specific value. Such a powerful querying mechanism is not available using available data querying methods on individual databases but requires a certain level of data unification and linking.

A data structure suitable for unifying and connecting heterogeneous data is a graph. The graphs are pairs of sets of nodes and edges, where nodes represent objects and edges represent relations between pairs of nodes. The flexibility of the data structure enables the wide use of graphs in a wide range of applications, from solving universal network-related problems [2,3,4] to molecular structure analysis [5] and machine learning models [6]. A special type of graph, containing semantic relations between the entities is known as a knowledge graph [7].

There are active initiatives to join data from multiple datasets into a knowledge graph. Most currently available solutions focus on particular subdomains, such as drug discovery and proteomics, but rarely on the overall connection of general biological data from various domains. The solutions mostly rely on RDF (Resource Description Framework) [8], which defines relations as subject-predicate-object triplets. One of the issues with RDF representation is the lack of the ability to provide properties of the relations. A gene may be related to a disease with a certain level of relation strength. The RDF format does not support any relation properties that would be used to define that relation strength value. An extended RDF* standard does support the relation properties, but most systems that use RDF do not always support the RDF* extension [9]. Querying complex data paths across multiple entities is not natively supported by RDF-based systems. On the other side, commercial solutions do not allow free access, which additionally limits the assessment of their functions. There are also approaches to define ontologies in specific biological subdomains, such as OBO Ontologies [10], but the data in many public databases do not follow those schemas.

This paper aims to present a new model which enables simultaneous querying of biological data properties from multiple datasets based on querying metadata available from the original databases. The model is not focused on copying the data from the original datasets but linking the metadata in a way that can be used for efficiently executing complex queries on linked data. The model allows adding properties to entities and relations and unifying metadata from diverse data formats. A tool that uses the new data model was also developed and made available as open source for further improvements and easy integration of new datasets by the community developers. The tool and the corresponding packages can be deployed locally as a standalone system so that the queries can be executed offline.

### Related Work

There is a broad range of knowledge graphs in the biomedical domain. The Biolink model [11] defines a data model for biological entities and is used in some open-source solutions, such as ROBOKOP (Reasoning Over Biomedical Objects linked in Knowledge-Oriented Pathways) [12]. The ROBOKOP system offers a knowledge graph of biomedical data that uses the Biolink model to represent high-level schemas. Monarch [13] is another open-source initiative with a knowledge graph solution based on relations between genotype and phenotype from different species and data sources. The system provides data querying by identifiers and neighborhood relations but does not enable querying patterns in graph data. Data loading is carried out using the Koza data transformation framework [14] maintained by the Monarch initiative [13]. GeneCard.org [15] provides tools for searching human-gene-centric data aggregated from more than 190 different data sources. Elsevier’s Biology Knowledge Graph [16] is a commercial, closed-source software solution designed to link biological data from multiple sources through manual annotation, automatic generation of links between data, and natural language processing of research papers. All the listed solutions have pros and cons, which will be discussed and compared against our proposed data model.

## 2. Results and Discussion

Current trends show that knowledge graphs are becoming more popular and widely used in many domains [17]. Using a knowledge graph for interconnecting data from biological data sources is not a novel idea. Knowledge graphs are the foundational structure for intelligent health care [18]. As mentioned in the related work section, many projects aim to transform a wide variety of biological data into knowledge graphs to capture the relations between objects in a convenient form and understand the deeper meaning of those relations. The meanings of relations are looked at from a biological point of view. However, they are discovered using computational methods powered by good data models and powerful tools that are easy to use. The graph data structure is a logical choice for implementing such data models. It is the most efficient structure for storing and querying highly interconnected data while allowing easy extensions without changing the model foundations. We have designed BioGraph, a new model that takes advantage of the graph structure while remaining as generic as possible to support different schemes defined at higher levels.

For any software system to be accepted by the research community, the availability of the system is the key property. Open-source and free-to-use systems are reaching a wider audience and providing their utility at a much higher degree than payware systems. On the other hand, deploying a sophisticated system on a platform available to researchers worldwide is unfeasible without any financial support. We decided to maintain the free availability of the system and its open-source properties by enabling the users to deploy the system components locally on their machines easily. The Monarch [13] and ROBOKOP [12] systems also follow the idea of open-source availability, while the Elsevier Biology graph [16] is a closed-source software.

The following important property for the broader adoption of a software system in the research community is simplicity. We aimed to create a simple, easy-to-use system that allows users with almost no technical know-how to express their data searching requests in the simplest possible way, enabled by the simplicity of the underlying data model. Simple data searching is enabled by graphical queries, where users draw the patterns between entities while also having the ability to run keyword searches to find specific entities that should be included in the search. Monarch allows users to run keyword searches but not pattern queries. The ROBOKOP [12] system allows drawing queries in graphical form but in a generalized and unintuitive way, making the system’s usage difficult for regular users. GeneCards.org [15] provides keyword-based search tools and does not support matching patterns between multiple entities. It also has support for queries using natural language. We decided not to support natural language queries in the initial version of the querying interface. Natural language queries, while being easy to use by the users, introduce unnecessary ambiguity in queries and uncertainty in results.

Extensibility is another key property. The easy extensibility of the data model and the overall software system is necessary for continuous development and upgrades of the system, not only by the authors but by the research community members. Our system and data model, being open-source, along with the easy addition of new import scripts, allow users to develop new scripts for importing data from countless data sources. Besides developing import scripts and other upgrades for themselves, developers can share their upgrades with other community members worldwide and further expand the adoption. Both Monarch [13] and ROBOKOP [12], as open-source systems, enable the extensibility of their systems.

It is often the case that the results of one research are inputs for the others. That is why saving the search results is also a feature of our system. Aside from saving the results, our system also allows saving and loading queries, so the researchers can share their queries and run them on different machines instead of transferring the results, which are orders of magnitude larger than the queries. The ROBOKOP [12] system also allows saving and loading queries from files, while Monarch [13] does not provide those features.

Comparison of the BioGraph system with similar projects for searching biological data using multiple criteria shows its advantages in use. A structured comparison between existing solutions and our model, with the corresponding querying system, is presented in Table 1. Most details about the Elsevier Biology Knowledge Graph [16] are missing due to its closed-source nature.

## 3. Methods and Materials

In this section, we present the proposed BioGraph data model, the methods we used to implement the model and the user-friendly interface, and the materials used to validate the implemented model.

### 3.1. Model Definition and Implementation

We have defined a set of goals that the ideal data model should fulfill so the integrated search of metadata sourced from various datasets could be achieved. Those goals are:

- Unified representation of metadata from diverse formats and schemas.
- Efficient querying and finding complex patterns in data.
- Allowing easy extensibility through schema extensions without affecting the core data model.
- Being database-agnostic as much as possible, so the underlying database management system and related software do not dictate the model’s design.

Biological data contain information about biological entities and their properties. We recognized those entities as the primary building blocks of the new model. Entities are not isolated objects but are highly interconnected in many ways. The relations between the entities have various origins, from biological processes to taxonomical relations and mutual similarities. Associating entities from multiple datasets are enabled by connecting relations paths between them across multiple datasets. As many types of entities exist, creating specific model schemas for each biological entity and their relations is inefficient. It violates the requirement of unified representation, and there is no feasible way of predicting all future properties and relations associated with every entity type. That is why we decided to maintain a model which enables storing metadata of arbitrary entity types in a generic form. Details regarding entity type-specific information are delegated to high-level data schemas. It is essential to mention that our model links metadata related to biological entities in a way that can be used for efficient data location and retrieval from the original data sources. An example of gene metadata might be its identifier, while the data are a DNA sequence representing the gene. The gene sequence is not handled by the model but can be easily retrieved from the external database, such as the NCBI gene, using the metadata linked in the model.

### 3.2. Model Definition

**Figure 1:**
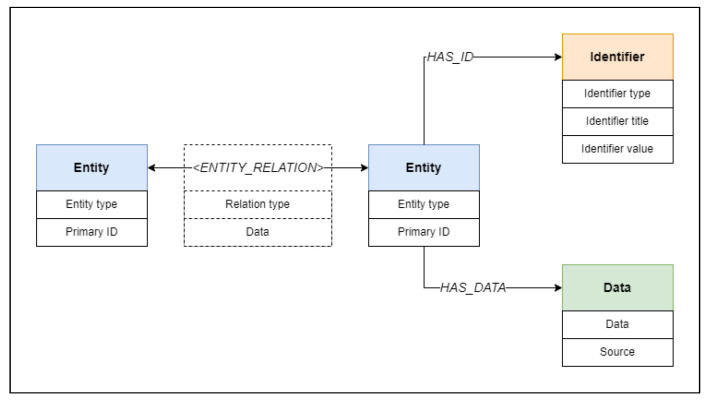
Base schema of the BioGraph model. Entities of specified types, identified using primary identifiers, have connections with all of their identifiers and data objects, as well as with other entities. The identifier objects contain information about the nature of the identifier and the identifier values, while data objects store metadata collected from datasets along with the label of the dataset source. Relations between entities are defined using the relation type and additional data that can further explain the nature and strength of the relationship.

Our model consists of three object types: entity objects, identifier objects, data objects, and relations that connect the objects. Model objects and their individual relationships are shown in Figure 1. Metadata are loaded from arbitrary data formats, from which entities, identifiers, and data objects are extracted along with respective relations. The extracted objects and relations form a unified knowledge graph ready for searching, having each object represented as a graph node connected to other nodes using the relation edges.

**Figure 2:**
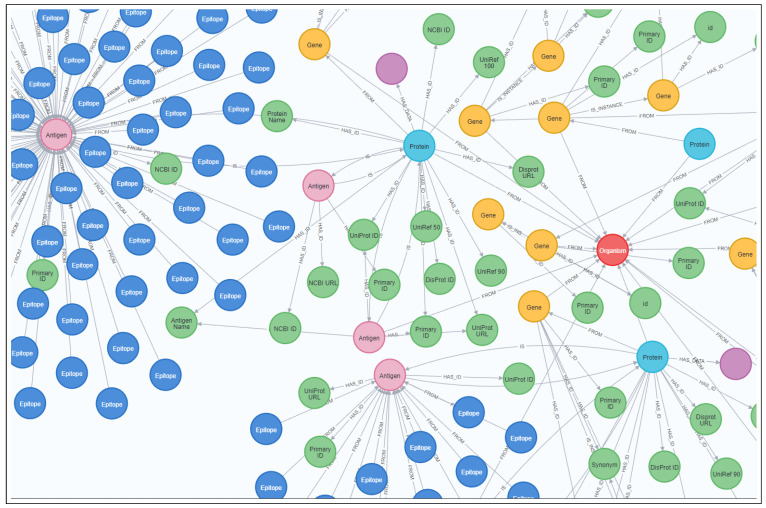
Segment of the knowledge graph constructed from BioGraph model objects and relations extracted from data imported from DisProt [19], DisGeNET [20], Tantigen 2.0 [21], IEDB [22], and HGNC [23] datasets. A red node represents an organism entity, green nodes represent identifiers, purple nodes represent data objects, yellow nodes represent gene entities, cyan nodes represent proteins, pink nodes represent antigen entities, and blue nodes represent epitopes.

Details about all created objects and relations are stored in a catalog database for additional indexing. Figure 2 shows a small segment of the constructed knowledge graph, displayed using the Neo4J browser.

Biological entities come in different types. Some are associated with biological functions, such as genes or proteins, while others can be more general, such as habitats and coverage areas. Regardless of the type, each entity can be assigned at least one unique identifier, which we denote as a primary identifier. When there are multiple unique identifiers, the primary identifier is chosen to be the one shared between most biological datasets. An example of a primary identifier for a gene entity is a gene name. It is important to note that the data model is not restricted to human genetic data, but can also support data from arbitrary biological domains regardless of the data source. We use entity type names and primary identifiers to construct entity objects in our data model. Each entity object represents a single biological entity.

**Figure 3:**
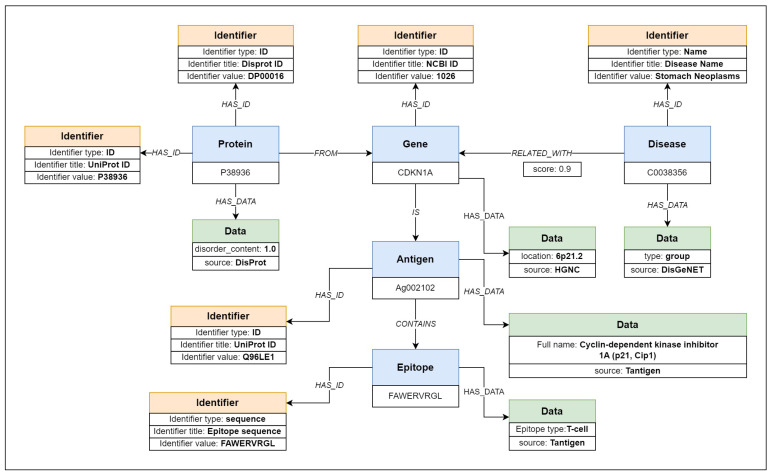
Network of objects from five different sources (DisProt [19], DisGeNET [20], Tantigen 2.0 [21], IEDB [22], and HGNC [23]) represented using the BioGraph model. The example shows protein data collected from DisProt [19] dataset, with identifiers assigned by DisProt and UniProt [24] along with disorder content value. Gene data, collected from HGNC [23] dataset, are connected with protein data from DisProt [19] dataset assigning the gene NCBI [25] identifier. Disease data from DisGeNET [20] are connected to the corresponding gene with an assigned relation score from DisGeNET [20] dataset. Tantigen 2.0 [21] and IEDB [22] datasets add context to the antigen nature of the gene, including information on the epitope of the antigen.

Figure 3 shows concrete examples of biological objects (sourced from DisProt [19], DisGeNET [20], Tantigen 2.0 [21], IEDB [22], and HGNC [23] datasets) and their relations. Five different entities are in blue boxes along with their type label and primary identifiers. Those entities, with their respective primary identifiers, are gene CDKN1A, protein P38936, disease C0038356, antigen Ag002102, and epitope with the sequence FAWERVRGL.

All available entity identifiers, including the primary identifier, are collected and represented in our model using identifier objects. The identifier objects are connected to their respective entity objects using identifier relations labeled as “HAS ID” relations. Each identifier contains information about the identifier type, title, and value. The identifier type defines the nature of the identifier—a name, a URL, or a generic identifier without any specific type. The identifier title is used to determine the meaning of the identifier, such as “gene name” or “NCBI ID”. Finally, the value stores the string value of the identifier. One identifier can be shared between multiple entities, so one identifier object can be connected to multiple entity objects. Besides identifiers, datasets may contain additional metadata about the entities, such as protein regions, location coordinates, or gene positions on chromosomes. We collect those metadata values and store them in data objects, along with the source label so that the metadata can be easily tracked and verified against the original dataset. Figure 3 show six identifiers in yellow boxes, connected to five entities. The example protein entity is connected with two identifiers, where P38936 is the identifier of the protein in the UniProt [24] database (also used as a primary identifier) and DP00016 is the identifier of the same protein in DisProt [19] database.

The data objects, containing the metadata of the entities, that are not identifiers, are associated with their respective entities using data relations labeled as “HAS DATA” relations. The example in Figure 3 shows data objects that give additional information to entities. A data object connected to a protein entity contains disorder content value, gene metadata contain gene location on chromosome, disease metadata contain disease type annotation, antigen metadata contain the full name of the antigen, and epitope data contain type annotation for the epitope.

Relations can also exist between entities. A protein can be associated with its source gene, and a blood sample can be associated with the laboratory where it was collected. As the entity relations are various, they are defined in high-level schemas. Base relations between entities are displayed in Table 2, but the list can be extended using higher-level schemas. All relations in our model share the same structure, containing information about the relation label and optional data specifying the details of a connection between the associated entities.

An example network of biological objects from five different sources (DisProt [19], DisGeNET [20], Tantigen [21], IEDB [22], and HGNC [23]) represented using the BioGraph model elements is displayed in Figure 3.

Duplicate entries can negatively affect search results and the overall performance of searching. Our model assigns specific identifiers to objects and relations based on their content to prevent duplicate entries. The method for generating such identifiers is called content addressing. Data stored in an object or relation are mapped to a number from a large interval using a hash function, with a very low probability of having multiple different data mapped to the same number. If the mapping is performed multiple times on the same object, it will always result in the same number, and the resulting number can be used as a unique identifier.

### 3.3. Model Implementation

We have tested multiple database systems for storing metadata objects. Although the model was successfully mapped to relation databases, querying speeds were not satisfying, so we decided to use a graph database system as the underlying database. The Neo4J [26] graph database system provided the best overall results, but the model is not restricted to a specific data storage system. We used a relational database as a catalog database to store the import metadata of all objects and relations, preprocessed text metadata for semantic searches, and entity identifiers for quicker entity lookups. Both graph and catalog databases are kept synchronized, and data modification is carried out exclusively in transaction mode.

The tool that implements the presented BioGraph model was created using Javascript programming language and NodeJS v19 runtime environment [27]. The tool enables transforming and importing metadata from different data sources to objects and relations described in the model, indexing the catalog of imported entries, and executing queries for searching the stored data. The set of scripts used for metadata importing and transformation is extensible, and developers can easily include their own scripts for processing metadata from various sources. All import scripts are connected to the core BioGraph service, which exposes five essential methods for extracting model objects and relations and importing metadata. The developers use the exposed methods to start imports, map the metadata segments to objects and relations, and finish the import. The system can be deployed locally as a standalone system so that the queries can be executed offline.

**Figure 4:**
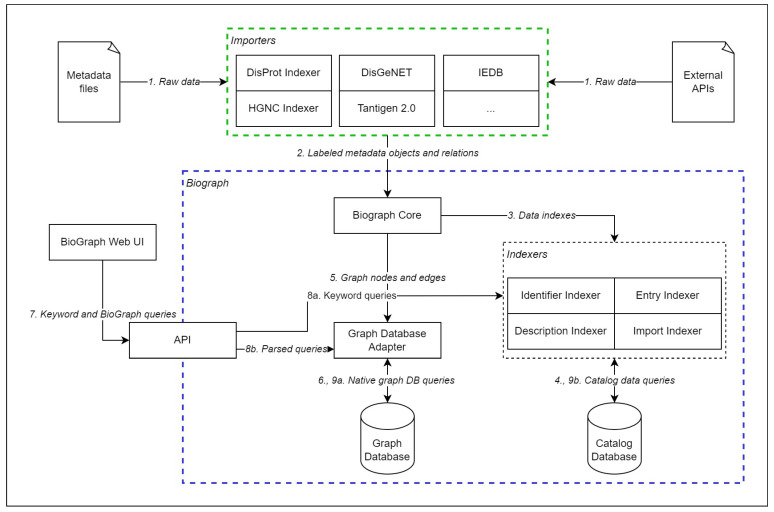
Diagram representing the architecture of the system which implements the BioGraph data model. The raw data are collected from external data sources (step 1) and a subset of collected data is labeled and transformed into BioGraph model elements using importers specialized for the selected external data source. The list of importers shown in the figure is not final; it represents the current state of the system and can be easily extended for different data sources. Labeled metadata and relations (step 2) are then sent to the BiogGraph core service, which prepares graph nodes and edges based on the BioGraph model elements and sends them to indexers for storing in the catalog database (step 3). Indexers individually log all objects into the catalog database (step 4), preventing duplicate entries. New, non-duplicate, entries are sent to the graph database adapter (step 5) which converts graph elements into storage queries in the native language of the underlying graph database and executes them (step 6). External applications, such as BioGraph Web UI, communicate with the BioGraph services using exposed API (step 7). The applications send queries in a form of the internal JSON query language. API sends parsed queries to either indexers (step 8a), in case of keyword queries, or graph database adapter (step 8b), in case of graph queries. Graph queries are executed on the graph database (step 9a) while keyword queries are executed on the catalog database (step 9b).

The BioGraph system consists of importers, indexers, database connectors, API, and a core library, which connects all the pieces into a working system. Importers transform import data into data model objects using the methods exposed from the BioGraph core library. The core library maps the abstract model objects into graph nodes and edges that are stored in the graph database with the help of a graph database adapter. The core library also sends information about the received imports and objects to indexers, which store the data in the catalog database. There are four types of indexers: entry indexer, identifier indexer, description indexer, and import indexer. The identifier indexer stores identifiers in catalog tables indexed for quick keyword lookups, while description indexers store text descriptions of the objects in a similarly indexed table. The entry indexer logs the information about all stored objects and relations, while the import indexer logs all imports and links to objects that were imported during specific imports. The API allows external programs to access the BioGraph data with the help of a query parser, which transforms BioGraph queries into native language queries of the underlying graph database. The API specification is listed in the software documentation while the entire architecture of the BioGraph system is displayed in Figure 4.

### 3.4. Data Flows

To give a complete picture of the BioGraph system, Figure 4 shows enumerated steps for data transformation and retrieval, from importing data into the graph and catalog databases to fetching the query results in the Web user interface. The central group of services, shown in Figure 4, enclosed in a blue dashed rectangle, is the core of the BioGraph system and it is static. Importers, enclosed in a green dashed rectangle, are dynamic services, that can be modified to fit any external data source. The first step of the process (step 1) is the collection of raw data from external databases. The data are downloaded using APIs of the external data sources, FTP access, or even scraping the web pages. The method for collecting the data is implemented into the import services, which can be customized for any data source. The subset of collected data is labeled within the import service and mapped to BioGraph objects and relations.

In the next step (step 2), labeled objects and relations go to the BioGraph Core service where the objects are first logged in the catalog database using indexers (step 3). Identifiers, descriptions, and general import information are indexed individually and stored in the catalog database (step 4). Duplicate entries are skipped, while non-duplicate objects and relations are sent to the graph database adapter (step 5). The graph database adapter transforms objects and relations into native storage queries of the underlying graph database and executes the queries on the graph database (step 6). New graph database adapters can be implemented for many of the existing graph database management systems. Storing both graph and catalog data is performed in transaction mode, to prevent data inconsistencies.

Once the data are stored in both graph and catalog databases, external applications, such as BioGraph Web UI can send BioGraph queries and keyword queries to the BioGraph API service in JSON form (step 7). The keyword queries are sent to indexers (step 8a) and executed on the catalog database (9b), while the parsed graph queries are transformed into native graph database queries in the graph database adapter (step 8b) and sent to the graph database (step 9b). Text data stored in the catalog database are internally stored in prefix trees to support the efficient execution of keyword queries.

The data flow once per import through the importers, with the exception of importing data updates, while the stored data can be queried arbitrarily many times using the core services.

### 3.5. Searching the Data

**Figure 5:**
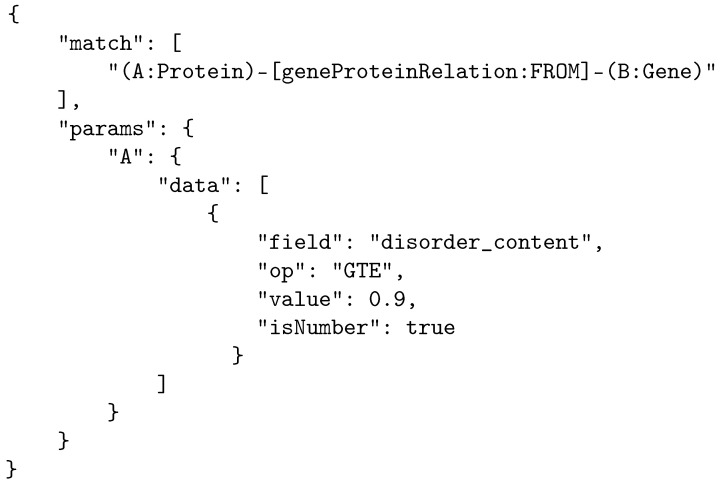
Example of an internal BioGraph query in JSON format. The query fetches all genes and related proteins where the protein disorder content is 0.9 or higher.

To remain database-agnostic, we designed a simple and generic internal query language that can be easily transformed into any of the native languages of the underlying database systems to support easy and efficient searches through metadata following the structure of the proposed model. The search is performed using query objects in JSON [28] format, divided into two segments: “match” and “params”. The “match” segment lists the relations between the searched entities, while the “params” segment lists the identifiers and attributes of the searched objects and relations. An example of a query using internal BioGraph query language is shown in Figure 5.

Users with technical experience can write their own applications to submit queries to BioGraph and use BioGraph as a backend system. Even though the integrated query language is simple and intuitive for users with technical backgrounds, it still may not be friendly enough for users with little or no technical experience. That is why we have also implemented a Web-based graphical user interface, developed using React framework, that communicates with BioGraph and can be used to create and execute graphical searches. It enables users to select predefined entity types and relations between the selected entities to draw a pattern that will be matched against the metadata graph. Graphical queries allow users without significant technical experience to intuitively create complex queries and discover indirect links between entities. Users can further explore the metadata of the entities from the query results and navigate through the graph following relations to neighboring entities. Additionally, the Web interface offers example queries and a help section to enable quicker onboarding of new users and a better understanding of the data retrieval process using graphical queries. All executed queries and results can be exported to files for further reuse and analysis. The file format for the exported queries is JSON, while the results can be exported in CSV format.

**Figure 6:**
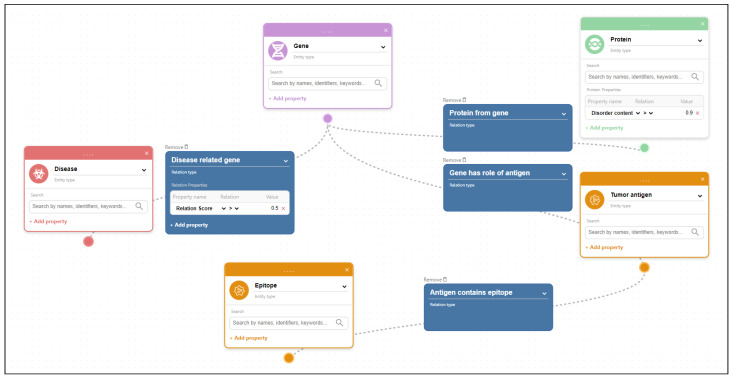
Example of a graphical query drawn using BioGraph Web interface. The data are fetched from imported metadata from the currently supported data sources. The query attempts to find links between Diseases and Genes with a DisGeNET [20] relation score of 0.5 and greater, where genes are transcribed into proteins dataset, with disorder content of 0.9 or higher and genes are also tumor antigens. Protein metadata come from the DisProt [19] dataset, gene metadata come from the HGNC [23] dataset, and disease metadata come from [20] dataset. Epitopes, from Tantigen 2.0 [21] and IEDB [22] are also fetched for the matched tumor antigens. The query pattern matches the structure of the example shown in Figure 3.

**Figure 7:**
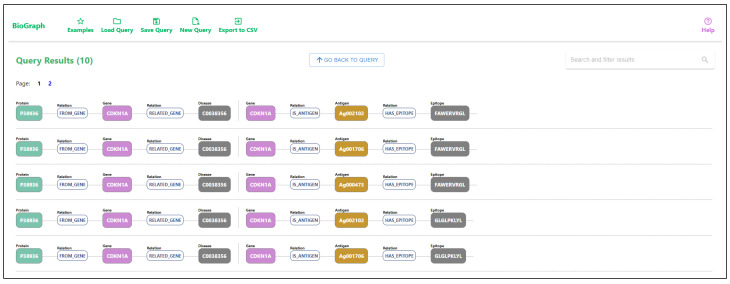
List of results received when querying the genes which are transcribed into highly disordered proteins (disorder content greater than or equal to 0.9) and linked with diseases with DisGeNET relation score of 0.5 and greater. We requested that the genes in the results are also tumor antigens and fetch all epitopes related to the antigens. The results are displayed as matched paths in the knowledge graph. The example shown in Figure 3 matches the first result in the list.

An example of a graphical query and the query results can be seen in Figure 6. The graphical query describes a pattern connecting genes and related diseases with a relation score greater than 0.5, where the genes are also related to proteins with a disorder content percentage greater than 0.9. The relation score between the gene and the disease is the DisGeNET gene-disease association score [20]. Additionally, we require that genes are also tumor antigens, and we want to match all epitopes related to those tumor antigens. Figure ref-results displays all matched patterns for the given graphical query. As the patterns are often not linear, we decompose the result patterns into linear disjoint paths. The results of the query are shown in Figure 7, where the first result matches the example shown in Figure 3.

**Figure 8:**
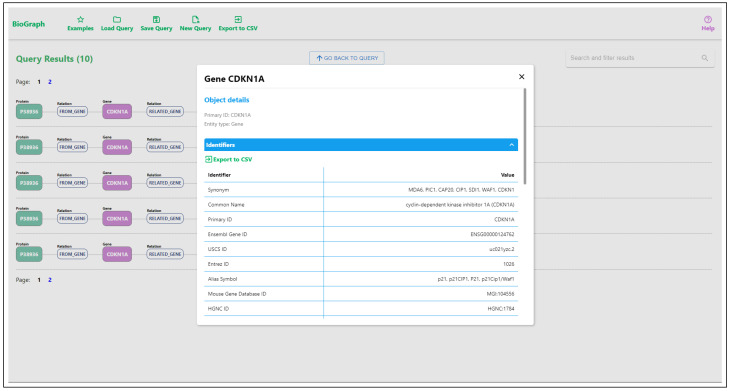
Details of a single gene entity, displaying information collected from multiple data sources.

**Figure 9:**
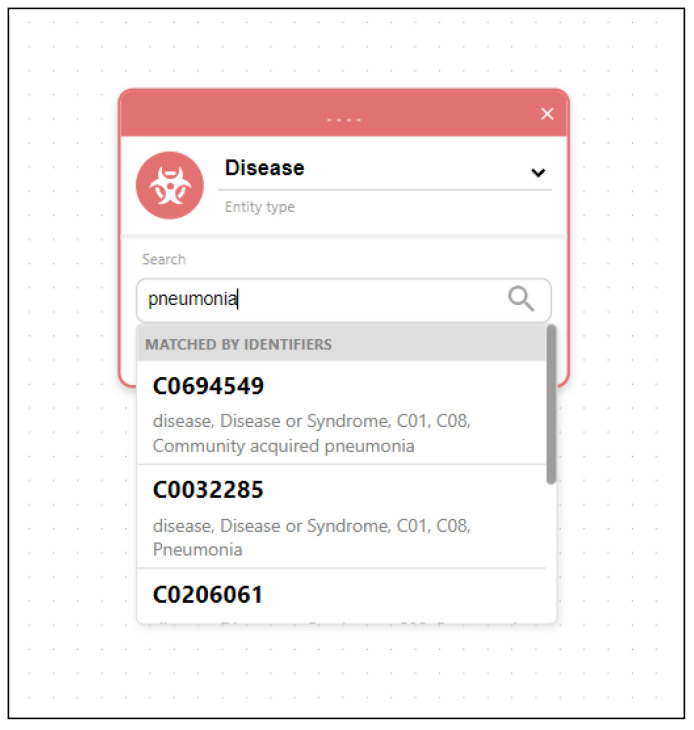
Searching diseases using keyword “pneumonia”.

Writing this type of query can be challenging in native database languages, while drawing a graphical pattern that the query should match is easy and intuitive. The results show paths from the matched patterns. Selecting any node from any path displays details about the specific entity and lists all the connected data and identifiers from all datasets. By following the relations listed in entity details, the user can easily traverse the graph and track the relations between the entities. An example of details for a specific gene is displayed in Figure 8. Besides using pattern matching, entities can also be searched using keyword searches through input fields located in entity nodes. An example of searching disease entity matching the keyword “pneumonia” is displayed in Figure 9.

The BioGraph service and the Web interface are deployable in the local environment on Linux, Windows, and macOS platforms, requiring the installation of NodeJS runtime and Neo4J DBMS. The source code for the web interface, along with the source code of the Biograph services, can be found at https://github.com/aleksandar-veljkovic/biograph (accessed on 25 February 2023). The predeployed BioGraph Web interface with all backend services preinstalled is currently available on http://andromeda.matf.bg.ac.rs:54321 (accessed on 25 February 2023), but we highly encourage deploying the system in a local environment to maintain offline data availability.

### 3.6. Materials

For verification of the proposed model and its implementation, we collected metadata from five diversely formatted biological datasets: DisProt [19], HGNC [23], IEDB [22], Tantigen 2.0 [21], and DisGeNET [20].

The DisProt (Database of Protein Disorder) dataset contains data on intrinsically disordered proteins from different species. The dataset currently consists of over 2300 protein entries. Metadata from the DisProt dataset were collected from DisProt API in JSON format.

The HGNC (HUGO Gene Nomenclature Committee, where HUGO stands for Human Genome Organization) dataset contains information on gene symbols, identifiers, gene chromosome locations, and their respective protein references for all known human genes. At the time of writing, the dataset contains 43,621 entries of human genes and also includes the identifiers of the ortholog genes found in mouse and rat genomes. Metadata from HGNC dataset were collected by downloading a JSON file.

The DisGeNet (Disease Gene Network) dataset contains information on the relations between genes and human diseases collected from multiple sources. The dataset consists of 1,134,942 gene-disease relation entries. The metadata from DisGeNET dataset were collected as a TSV document.

The IEDB (Immune Epitope Database) dataset contains data on immune epitopes from different organisms. Each epitope is associated with its source antigen, including identifier references to both protein and non-protein antigens, and source organism taxon. Although the dataset contains more information, such as those related to diseases, we decided for our model verification to use only the subset of information containing the relations between epitopes and antigens. The dataset metadata were downloaded from the project website in the TSV format.

The Tantigen 2.0 dataset contains data on human tumor antigens containing HLA ligands and immune T-cell epitopes. The database website does not provide API access or downloadable files. Our importer scraped the content of the project website and transformed the collected HTML pages into JSON documents as a preprocessing step. The total number of antigen entries in the Tantigen 2.0 dataset at the time of writing is 4297.

**Figure 10:**
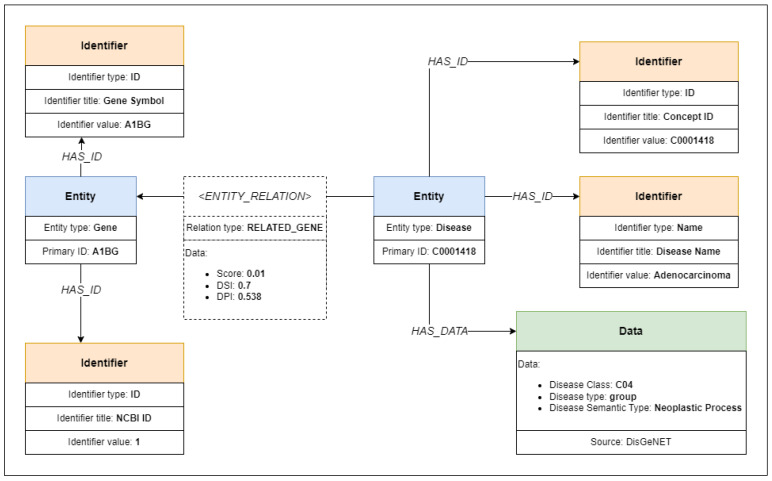
Diagram representing metadata from DisGeNET dataset record mapped to BioGraph model.

We have successfully transformed and imported and connected metadata from all datasets. The extracted metadata contained more than 16 million model objects, of which more than 2,500,000 individual entity objects, interconnected with more than 21 million relations. An example of the mapping of metadata from the DisGeNET dataset is provided in Figure 10.

The system may be applied to new transcript descriptions in the databases for model plant species [29,30]. Querying the biological databases via web-interface was recently presented in the ANDDigest module integrated into the ANDSystem tool, designed to search for information in pre-processed texts [31,32]. The ontology of the ANDSystem tool features dictionaries for molecular-genetics entities (genes, proteins, metabolites, microRNAs), cells and organisms [32], similar to our tool.

We have deployed the BioGraph services and the Web Interface Application to an Intel Xeon 3.50 GHz server with 16GB RAM running Windows 10 Server 2022 and Neo4J Community edition service. The data from the five datasets were preprocessed and imported into the graph and the catalog database in 10 h on the given machine. On a desktop workstation with 2.3 GHz Dual-Core Intel Core i5 and 8 GB RAM under MacOS Monterey, preprocessing and import time took 11 and a half hours. Still, the graph queries and searches by identifiers and keywords are executed in almost real-time (1–10 s), depending on query complexity.

## 4. Conclusions

The presented model enables information retrieval using interlinked entities from highly diverse biological datasets in a simple and unified manner. The model has advantages over similar models, from the simplicity of querying and the possibility of metadata integration from new data sources to the efficiency of metadata retrieval from complexly interconnected entities, which would be difficult to detect without interconnecting data. The promising results encourage further development of the model and the corresponding software systems while allowing community engagement in all improvement fields. Further research will focus on definitions of high-level metadata schemas and generating graph embeddings to discover semantic similarities between entities.

## Figures and Tables

**Table 1:** Comparison between the BioGraph system and currently existing solutions. The BioGraph system fulfills all the given criteria, except natural language queries, when compared to similar systems. The question marks in the table represent the states where the criteria could not be evaluated.

Criterium	BioGraph	ROBOKOP	Monarch	GeneCards	Elsevier Biology KG
Open-source	✓	✓	✓	✗	✗
Local deployment	✓	✓	✓	✗	✗
Pattern querying	✓	✓	✗	✗	?
Graphical queries	✓	✓	✗	✗	?
Extensibility	✓	✗	✗	?	?
Natural Language Queries	✗	✓	✗	✗	?
Loading and Storing Queries	✓	✓	✗	✗	?
User-friendly	✓	✗	✓	✓	?

**Table 2:** Base types of the relations between entity objects.

Relation	Description	Example
IS	Relation representing equality between objects, where object A, on one side of the relation can also be represented as B in general or specific circumstances. The relation can contain details specifying the equality relation.	Gene A IS Antigen B
IS INSTANCE	Relation between objects where one of the objects is an instance of a bigger class.	TP53 protein in humans IS INSTANCE of TP53 protein.
IS VARIANT	Representing relation between objects where one object is an isoform of the other.	Protein A IS VARIANT of protein B
FROM	Represents the relation between the object and its source.	Protein A FROM gene B. Gene C FROM organism D
CONTAINS	Represents the relation between the object and its part.	Antigen A CONTAINS Epitope B
RELATED WITH	General relationship between objects. A weight, or relation score, of the relation can be defined in relation parameters.	Gene A is RELATED WITH disease B, with a relation score of 0.9

## Data Availability

Not applicable.

## Abbreviations

The following abbreviations are used in this manuscript:

API	Application Programming Interface
CSV	Comma-Separated Values
DisGeNET	Disease Gene Network
DisProt	Database of Protein Disorder
HGNC	HUGO Gene Nomenclature Committee
HUGO	Human Genome Organization
IEDB	Immune Epitope DataBase
JSON	JavaScript Object Notation
KG	Knowledge Graph
NCBI	National Center for Biotechnology Information
OBO	Open Biomedical Ontologies
RDF	Resource Description Framework
ROBOKOP	Reasoning Over Biomedical Objects linked in Knowledge Oriented Pathways
TSV	Tab-Separated Values
UniProt	Universal Protein Resource
